# Editorial: Celebrating the Diversity of Genetic Research to Dissect the Pathogenesis of Parkinson's Disease

**DOI:** 10.3389/fneur.2021.648417

**Published:** 2021-04-06

**Authors:** Matthew J. Farrer, Soraya Bardien, Nobutaka Hattori, Suzanne Lesage, Owen A. Ross, George D. Mellick, Rejko Kruger

**Affiliations:** ^1^Department of Neurology, University of Florida, Gainesville, FL, United States; ^2^Division of Molecular Biology and Human Genetics, Faculty of Medicine and Health Sciences, Stellenbosch University, Cape Town, South Africa; ^3^Department of Neurology, Juntendo University School of Medicine, Tokyo, Japan; ^4^Institut National de la Santé et de la Recherche Médicale U1127, Centre National de la Recherche Scientifique Unité Mixte de Recherche 7225, Sorbonne Université, Institut du Cerveau et de la Moelle épinière, ICM, Paris, France; ^5^Departments of Neuroscience and Clinical Genomics, Mayo Clinic, Jacksonville, FL, United States; ^6^Griffith Institute for Drug Discovery, Griffith University, Nathan, QLD, Australia; ^7^Luxembourg Centre for Systems Biomedicine (LCSB), Esch-sur-Alzette, Luxembourg; ^8^Centre for Systems Biomedicine (LCSB), Belvaux, Luxembourg; ^9^Centre Hospitalier de Luxembourg (CHL), Luxembourg, Luxembourg

**Keywords:** Parkinson's disease, genetic etiology, disease progression, geographic diversity, GEoPD consortium

## Introduction

Parkinson's disease (PD) is the fastest growing neurological disorder worldwide, taking into account age-standardized rates for prevalence, disability and deaths (1). PD is characterized by a clinical symptomatology involving both motor and non-motor symptoms. According to the Global Burden of Disease study (2018), the global burden of this disorder has more than doubled over the past two decades from 2.5 million patients in 1990 to 6.1 million patients in 2016 (2).

In this editorial and eBook, we highlight the research done on PD by members of a global consortium known as the Genetic Epidemiology of Parkinson's disease (GEoPD) Consortium. We begin the editorial by providing a brief history of how GEoPD was started and how it has subsequently developed into an international endeavor. We then briefly summarize the completed and ongoing projects, and conclude with the future vision of this unique consortium.

## From Friends on a Road Trip to an International Roadmap for Solving the Puzzles of PD

GEoPD is a group of researchers dedicated to promoting education, scientific research, and translational development in PD. It is the longest running worldwide Consortium on PD, operating since 2004, and initially funded by a Michael J. Fox Foundation award to form “global genetic consortia.” GEoPD enables unfettered access to a “family” of multi-disciplinary expertise, including specialty neurologists, geneticists, biologists, epidemiologists or statisticians. From its inception, GEoPD has always maintained its tradition of diversity and inclusion with an active and growing membership from more than 60 sites and 30 countries on six continents.

The democratization of data, resources, projects and funding are long-established principles. To ensure open, honest collaboration and transparency without politics and control, GEoPD has always maintained an elected leadership. The GEoPD President is elected from and by a Steering Committee, for which each member serves a 2-year term that is renewable. Election to the Steering Committee is based on past contributions to the Consortium including: (1) directing a collaborative project, and/or; (2) directing a Core service (as specified below), and/or; (3) hosting the annual meeting.

Participation and projects are funded by and collectively for the members, generally through grants and sponsorships from different funding agencies and national societies. Projects include whole exome and genome sequencing, custom array genotyping, and studies that range from longitudinal assessment of idiopathic PD to monogenetic parkinsonism in families, to analyses of genetic and environmental variables using a Mendelian randomization approach.

**Figure 1:**
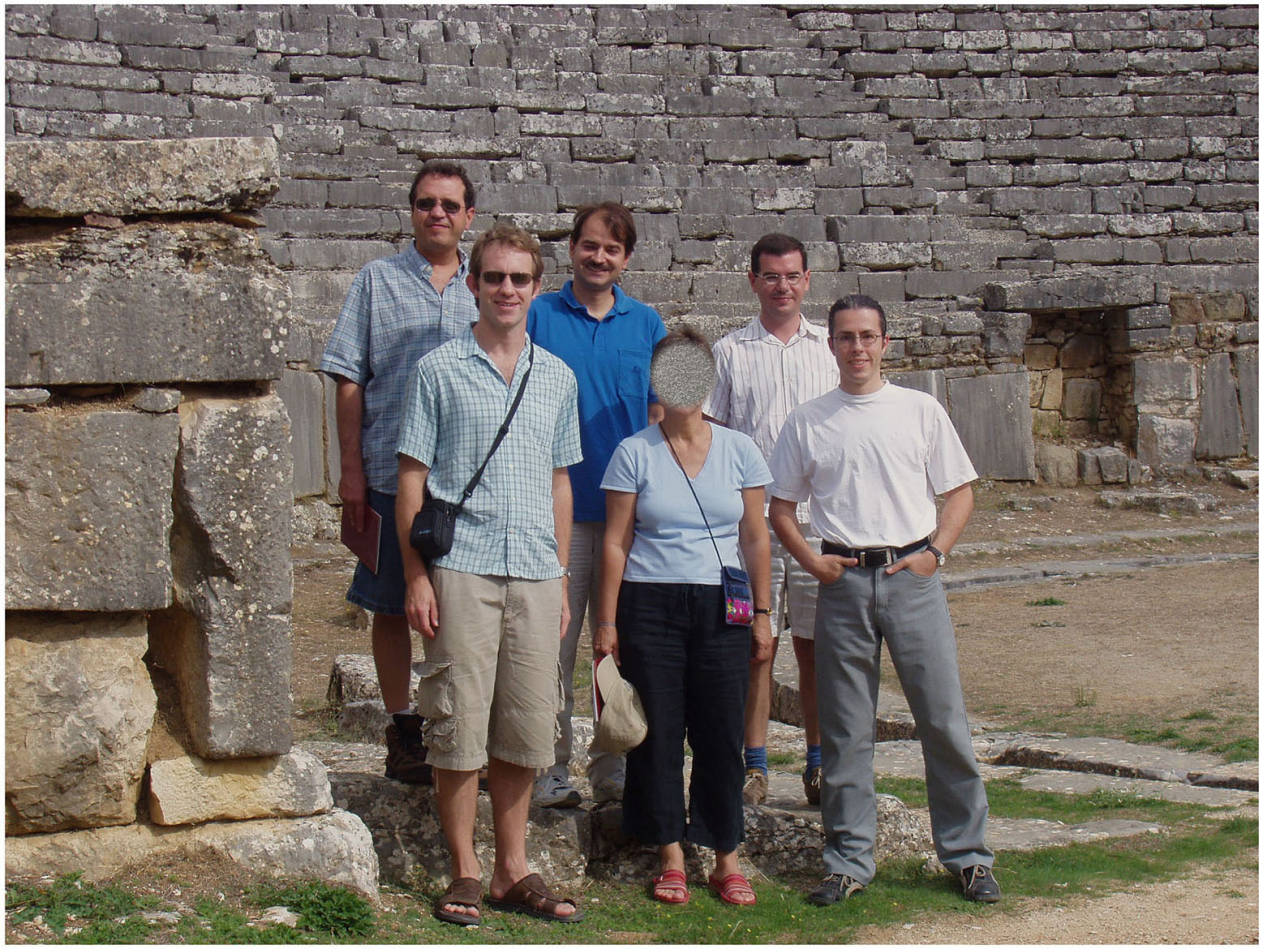
Founding members of GEoPD in Greece in 2004. Permission for publishing this figure has been obtained from the five individuals shown.

The first GEoPD meeting was organized by Demetrius (“Jim”) Maraganore in 2004 in Greece and was attended by six investigators that included Alexis Elbaz, Matt Farrer, and Rejko Kruger, who remain active members of the Consortium as Core leaders, and as past and current GEoPD Presidents. The six of them drove in a minibus from Athens, in the southeast, to Ioannina in the northwest, *via* Patras (Figure 1). Their tour guides on this fateful journey were Jim Maraganore and John Ioannidis. In part, the mission was to visit the ancestral origin of the alpha-synuclein p.A53T mutation, the first genetic mutation to be associated with familial late-onset parkinsonism. Focused on PD and genetic epidemiology, it was a remarkable journey of scientific discovery. They jointly elected to share that journey with likeminded colleagues around the world and Rejko Kruger coined the name “GEoPD” on the minibus. Ever since, one member of the Consortium has elected to host the meeting in a different part of the globe.

The aptly described GEoPD “World Tour” is CME-accredited and provides a forum where colleagues catch up, in person and/or virtually, and discuss their work and the latest developments in PD research. It is an educational opportunity that openly shares unpublished data and insights, that engagingly debates controversy in the field, and in a convivial setting. A central part of the meeting is reserved for a “data blitz” session that gives members ~10 min to highlight unpublished data and seek the help and collaboration of the entire membership. As a first exemplary project, GEoPD assessed the role of alpha-synuclein beyond autosomal dominantly inherited PD and established common regulatory polymorphisms in the *SNCA* gene as a risk factor for sporadic PD worldwide (3). This first global confirmation of *SNCA* as a risk factor in sporadic PD was subsequently confirmed by an unbiased approach in the era of genome-wide association studies (GWAS) (4).

## Developing a Truly International Collaboration

GEoPD is currently organized into five cores, each having a specific mandate about one of the following areas: bioinformatics, biology, clinical, communications, epidemiology, and statistics. The consortium's main mission is to promote multi-investigator research projects. Annual Meetings, held since 2005, offer a valuable forum for consortium members to discuss unpublished data and ideas, highlight research questions or needs, and identify global opportunities for partnership. These in-person meetings are organized and hosted each year in a different country by one of the members; the first “official” meeting was organized in Paris, France in 2005, and subsequent meetings took place in Santorini (Greece, 2006), Trondheim (Norway, 2008), Tübingen (Germany, 2009), Toronto (Canada, 2010), Evanston (USA, 2011), Seoul (South Korea, 2012), Lübeck (Germany, 2013), Vancouver (Canada, 2014), Tokyo (Japan, 2015), Luxembourg (Luxembourg, 2016), Cairns (Australia, 2017), Paris (France, 2018), Cape Town (South Africa, 2019), and in 2020 the first virtual meeting (due to the Coronavirus-2019 pandemic) hosted by the group in Milan, Italy.

Using the large multi-ethnic clinical and genetic datasets (currently including over 40,000 PD patients and 40,000 controls, mainly of European and Asian origins), multiple advanced analyses are performed to assess emerging mutations or variants associated with PD, and several studies have been published. We have examined the role of ~120 *LRRK2* coding variants in ~15,000 individuals, to implicate frequent substitutions in idiopathic PD and neuroprotection (5). Additionally, interactions between *LRRK2* and *PARK16 (RAB7L1; RAB29)* variants were not replicated in GEoPD efforts (6). Similarly, we have not been able to provide any evidence of an interaction of LRRK2 p.R1398H, which has a protective effect, with *MAPT* or *SNCA* variants (7). We have also questioned the role of intermediate size repeat expansions in *SCA2, SCA3, SCA6*, and *SCA17* (8), or *C9orf72* expansions (9), as risk factors for idiopathic PD and our findings excluded a major role of any of these intermediate/expanded repeats in PD pathogenesis. Overall, the GEoPD consortium has contributed more than 20 original, globally collaborative articles to advance our understanding of the genetic architecture of PD [(3, 5–23), Markopoulou et al.; Rajan et al.]. Recently, a unique global initiative from our consortium aims to identify all patients and relatives with *SNCA* multiplications to inform alpha-synuclein targeted therapeutic development (22). Longitudinal clinical assessments, genealogic information, genotyping data, and *SNCA* locus breakpoints from 59 families with *SNCA* multiplications are publicly available via a website that has been created as a forum for data exchange.

## Ongoing Projects

Details about the ongoing collaborative projects of the consortium can be found at the GEoPD website (https://www.geopd.net/projects). These include: **Monogenic PD** (a project to collect clinical and genetic information on mutation-positive monogenic PD individuals to inform genotype-phenotype correlations (21); **LONG-PD** a prospective study to assess disease progression, treatment response and outcomes in a longitudinal manner over >10 years in different ethnic cohorts of PD patients; **Courage PD** (COmprehensive Unbiased Risk factor Assessment for Genetics and Environment in Parkinson‘s Disease); **RVCD** (a study identifying rare sequence variants segregating in Mendelian forms of PD); and the **Trios** project, which aims to study PD-affected individuals and both of their biological parents using whole exome sequencing.

## The Future Vision of GEoPD

Over the past two decades, GEoPD has significantly contributed to the genetic dissection of PD, and established genetics as an entry point to decipher molecular mechanisms underlying neurodegeneration in this increasingly common age-related disorder. The mission of GEoPD is to inspire and unite researchers, at a global scale, to advance multidisciplinary research on genetic and environmental causes of PD, and to share insights and resources to enable translational neuroscience and clinical applications. The democratically elected structure of GEoPD allows direct and equal participation of all member sites worldwide; intellectual scientific contributions to the GEoPD enterprise, publicly voiced, discussed and ratified by the membership, are then supported by the efforts of the collective. Hence, the science is neither convened nor limited by the desires and constraints of funding agencies, nor political influence. This concept provides assurance that each site can jointly own and participate in federated data and sample infrastructure. Furthermore, this vision continues to draw new members from countries whose populations remain underrepresented in worldwide research, to promote their research in a global effort.

The advance of large-scale genotyping technologies and high-throughput sequencing has not yet directly translated into corresponding advances in elucidating the missing heritability of PD. New strategies are required to disentangle the complex genetic architecture of PD, to establish the molecular pathogenesis of this disorder, and to inform therapeutic development. To date, most genetic discoveries have been made in Caucasians of European ancestry, but these populations do not include the genetic diversity of different ethnicities worldwide. Identifying new genes, consolidating candidate genes and defining the impact of genetic variability in diverse populations has been a priority for GEoPD since its inception. This is underscored by the outreach to underrepresented populations at the first African GEoPD meeting in 2019 which served as the basis for this Frontiers eBook.

To fully capitalize on opportunities for genotype-phenotype correlations, GEoPD has addressed the emerging need for deep clinical phenotyping in PD and control cohorts (i.e., to improve stratification of patient heterogeneity, inform prognosis and enhance clinical trials). This is reflected by our efforts to harmonize clinical data-capture across GEoPD sites worldwide [e.g., in the LONG-PD study (led by Katerina Markopoulou) or Minimal Dataset initiatives available to all sites *via* the Elixir node for translational medicine data in Luxembourg].

Our understanding of genotype-phenotype correlations is currently limited due to a lack of systematic assessment of the functional role of novel gene regulatory variants and splicing defects (e.g., *via* differential regulation of gene expression). The increasing number of novel PD genes and risk variants being identified further underscores the need for functional validation of novel mutations using models to define disease-relevant molecular pathways. “Mechanism-based” stratification of molecular heterogeneity will inform genetically-stratified patient participation in clinical trials, and the first targeted treatment options (i.e., precision medicine). In this context, patient-based induced pluripotent stem cells (iPSC) including isogenic controls, provide an essential tool to study different disease-related variants in defined genetic backgrounds.

While rare mutations in monogenic forms of PD pave the way for precision medicine, the biological pathways impacted can reveal generic mechanisms that apply to larger groups of idiopathic patients (24). Many more genes have been revealed by genome-wide association meta-analyses that assess frequent, polymorphic variants of minor effect, although that polygenic risk is not predictive (25). However, recent innovations in whole genome sequencing, distributed cloud computing and artificial intelligence promise far greater breakthroughs in medical research discovery. We now have the opportunity to define the joint contribution of all genetic variants, including those of intermediate frequency and modest effect, provided that sample sizes are sufficiently large. The collaborative spirit of the GEoPD consortium strongly supports these larger scale international initiatives. Hence, we have embraced the Global Parkinson's Genetics Program (GP2) effort, led by Andrew Singleton, supported by the US National Institutes of Health and the recent Aligning Science Across Parkinson's (ASAP) initiative, that provides the opportunity to synergize and maximize the precious research contribution of people worldwide, with and without PD.

The 21 articles appearing in our first eBook in “Frontiers in Neurology: Neurogenetics Research Topic” highlights the breadth and depth of our scientific inquiry, and come from researchers working in Africa, Asia, Australia, Europe and North America. This reflects our expanding international collaborative effort, including the elected host site for the next GEoPD Annual Meeting in Omsk in southwestern Siberia in October 2021.

We welcome new members to join us by visiting our website at https://www.geopd.net/component/users/?view=registration.

## Author Contributions

GDM, SB, and OAR conceptualized the Editorial. All authors participated in drafting, writing, and reviewing the text.

## Conflict of Interest

The authors declare that the research was conducted in the absence of any commercial or financial relationships that could be construed as a potential conflict of interest.
